# *Linguatula serrata* (Porocephalida: Linguatulidae) Infection among Client-Owned Dogs in Jalingo, North Eastern Nigeria: Prevalence and Public Health Implications

**DOI:** 10.1155/2014/916120

**Published:** 2014-03-17

**Authors:** Oseni Saheed Oluwasina, Onyiche Emmanuel ThankGod, Omonuwa Omojefe Augustine, Fufa Ido Gimba

**Affiliations:** ^1^Department of Biological Sciences, Florida Atlantic University, 777 Glades Road, Boca Raton, FL 33431, USA; ^2^Department of Veterinary Microbiology and Parasitology, University of Agriculture, Abeokuta, Abeokuta 2280, Nigeria; ^3^Department of Veterinary Microbiology and Parasitology, University of Ibadan, Ibadan 200284, Nigeria; ^4^Department of Veterinary Public Health and Preventive Medicine, University of Ibadan, Ibadan 200284, Nigeria; ^5^Taraba State Veterinary Hospital, FGGC Road, Jalingo, Nigeria; ^6^Department of Veterinary Pathology and Microbiology, Universiti Putra Malaysia, 43400 Serdang, Darul Ehsan, Malaysia

## Abstract

Pentastomiasis is a parasitic zoonosis endemic to western and central Africa. This study was undertaken to determine the prevalence and public health implications of Linguatulosis in client-owned dogs in Jalingo, North Eastern Nigeria. Seven hundred and seventy seven (777) dogs brought for treatment at the hospital were subjected to buccal (sublingual) examination for pentastomiasis. Parameters such as age, sex, and breeds were determined. Also, the months of the year were taken into consideration. An overall prevalence of 37.45% was recorded. Of the 477 dogs examined in 2010, 184 were positive representing prevalence of 38.57% and in 2011 107 were positive representing prevalence of 35.67%. The infection was higher in the male than in the female which does not differ significantly (*P* > 0.05). There was no significant difference between sexes (*P* > 0.05). However, significant difference (*P* < 0.05) was observed between breeds and age of dogs examined. Season did not have much influence on the prevalence of Linguatulosis. The high prevalence of Linguatulosis in dogs and other animals found in this study highlights the need of improving preventative measures to reduce the rate of infection, which may pose a hazard to human health.

## 1. Introduction

Linguatulosis is a rare zoonotic parasitic disease resulting from invasion of the body by wormlike parasites of the genus* Linguatula*, also known as tongue worms [1]. The most commonly reported species involved in Linguatulosis is* Linguatula serrata* (family Linguatulidae, order Porocephalida, and phylum Pentastomida), which is commonly classified between annelids and arthropods [2].

Two families, Linguatulidae and Porocephalidae of which two important genera* Linguatula *and* Armillifer*, respectively, have been known to be of importance in veterinary and human medicine [3]. The species* Linguatula serrata* are enigmatic group of aberrant, worm-like, bloodsucking, zoonotic, and obligate endoparasites [4, 5] that inhabit the upper respiratory tract of terrestrial, carnivorous vertebrates.

*Linguatula serrata* are commonly called tongue worm, due to its characteristic transparent tongue-shaped, slightly convex, and ventrally flattened body structure [6]. The adult form of this parasite inhabits the nasal airway, frontal sinus, and tympanic cavity of canids and felids [7]. The intermediate hosts of these parasites are usually sheep, cattle, or rodents in which Visceral Linguatulosis have been described. The parasite is the most commonly reported pentastomid parasite of dogs and it corresponds to over 99% of reports from pentastomid infection [8]. In most cases, the parasites were detected at surgery or at autopsy, mainly in the liver, lungs, and lymph nodes. Because of the absence of specific clinical symptoms in parasitized dogs, diagnosis of Linguatulosis is often difficult.

Humans can be infected in two ways: either as an intermediate host (Visceral Linguatulosis) or on rare occasions as an accidental final host (Nasopharyngeal Linguatulosis), with the former being more frequently described [8]. Close contact to dogs and their secretions predispose to infection with* L. serrata* [9]. The highest prevalence of Visceral Linguatulosis due to* L. serrata* has been reported from the Middle East, where high infection rates for dogs have also been noted. Studies have shown that* L. serrata* was found in 43.3% of stray dogs in Beirut, Lebanon, 38% in parts of India, and in a high percentage in Mexico City [10].* L. serrata* in canines and humans can also lead to Nasopharyngeal Linguatulosis.

To the best of our knowledge, this is the first report of Canine Linguatulosis in Taraba State, North East of Nigeria. Other pentastomids (*Armillifer* sp.) have been reported in royal pythons [11] in the south western part of the country. Therefore, this study was carried out in the State Veterinary Hospital, Jalingo, Taraba State, Nigeria, in pet dogs brought for treatment. Risk factors and public health implications of Linguatulosis are also discussed in this study.

## 2. Materials and Method

**Figure 1:**
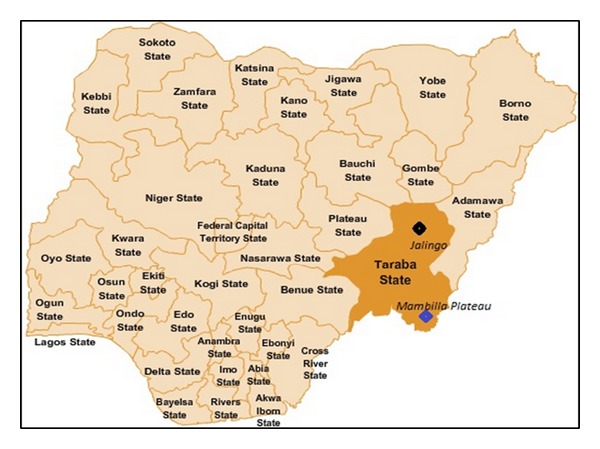
Map showing the study location in Taraba State, North Eastern Nigeria.

The study was conducted at the State Veterinary Hospital, Jalingo, Taraba State, Nigeria (8°00′N 10°30′E), between January 2010 and December 2011. Taraba covers an area of 60,291.8 square kilometers (Figure 1). It has a population of approximately 2,300,736 (2006 census figures).

The Mambilla Plateau with an altitude of 1,800 meters (6000 ft) above sea level has a temperate climate all year round. Seven hundred and seventy seven (777) dogs brought for treatment at the hospital were subjected to buccal (sublingual) examination and also observation for symptoms of pentastomiasis. Parameters such as age, sex, and breeds were determined. Also, the month of the year was taken into consideration. Those that harbored the parasite were subjected to a minor surgery to remove the parasite. This procedure is usually bloodless and takes less than 5 minutes. Recovered adult Linguatula parasites were flattened, dehydrated in ascending grades of ethyl alcohol, and cleared in creosote before examining under low power objective of microscope. The parasites were identified based on Soulsby [12] as* Linguatula serrata*.

## 3. Statistical Analysis

Statistical analysis was performed using chi-squared test. Significant level was set at *P* < 0.05.

## 4. Results (Figures 2–4)

The results of the prevalence of* Linguatula serrata* in dogs in Jalingo, North Eastern Nigeria, are presented in Table 1.

The body of adult* Linguatula serrata* parasite recovered was flat, elongated, annulated, and tongue like, and the anterior end had two pairs of hooks. The posterior extremity is somewhat narrow and cylindrical. The male worms measured about 17–20 mm in length and 2-3 mm in width. The females were 87–140 mm long and had a maximum width of 10–12 mm.

An overall prevalence of 37.45% was recorded. Of the 477 dogs examined in 2010, 184 were positive representing prevalence of 38.57%. The infection was higher in the male 124 (41.19%) than in the female 60 (34.09%). There was no significant difference between sexes (*P* > 0.05). Based on breed of dogs, out of the 350 local breed examined, 161 (46%) were positive followed by 21 (21%) and 2 (7.41%) of the 100 and 27 cross and exotic breeds of dogs, respectively. Differences were statistically significant (*P* < 0.05). Based on age, of the 259 dogs examined between 0–10 weeks, only 130 (50.19%) were positive while 54 (24.77%) of the 218 dogs examined between 11–20 weeks were positive. Significant differences were also observed across age groups (*P* < 0.05).

Of the 300 dogs examined in 2011, 107 were positive representing prevalence of 35.67%. The infection was higher in the male 67 (39.41%) than in the female 40 (30.77%). No significant difference was observed (*P* > 0.05). Based on the breed of dogs, of the 201 local breed of dogs examined, 102 (50.75%) were positive, followed by 2 (3.39%) of the 59 cross breeds of dogs examined, and finally 3 (7.5%) of the 40 exotic breeds of dogs examined were positive. Significant difference was observed (*P* < 0.05). Based on age, of the 161 dogs examined between 0–10 weeks, only 80 (49.69%) were positive and 27 (19.42%) were positive of the 139 dogs examined between the age range of 11–20 weeks old and differences were statistically significant (*P* < 0.05).

**Figure 2:**
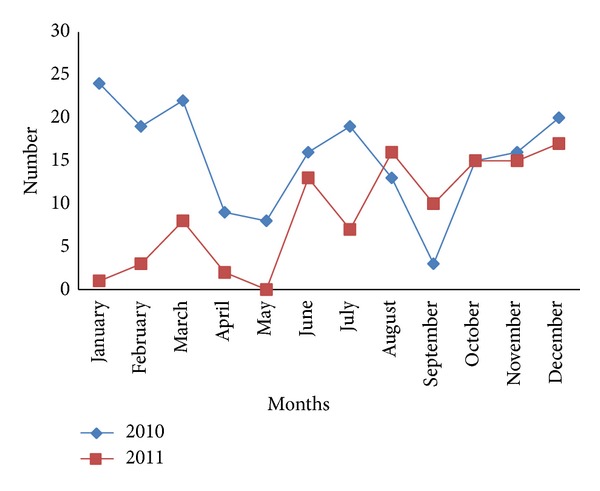
The monthly distribution of Linguatulosis in dogs in Jalingo, North Eastern Nigeria, for the years 2010 and 2011.

The seasonal prevalence of Linguatulosis increases from January to March. Just before the start of the rainy season in April to May, there was a sudden drop in the prevalence. This later picks up and increases from June till the end of the year in December (Figure 2).

**Figure 3:**
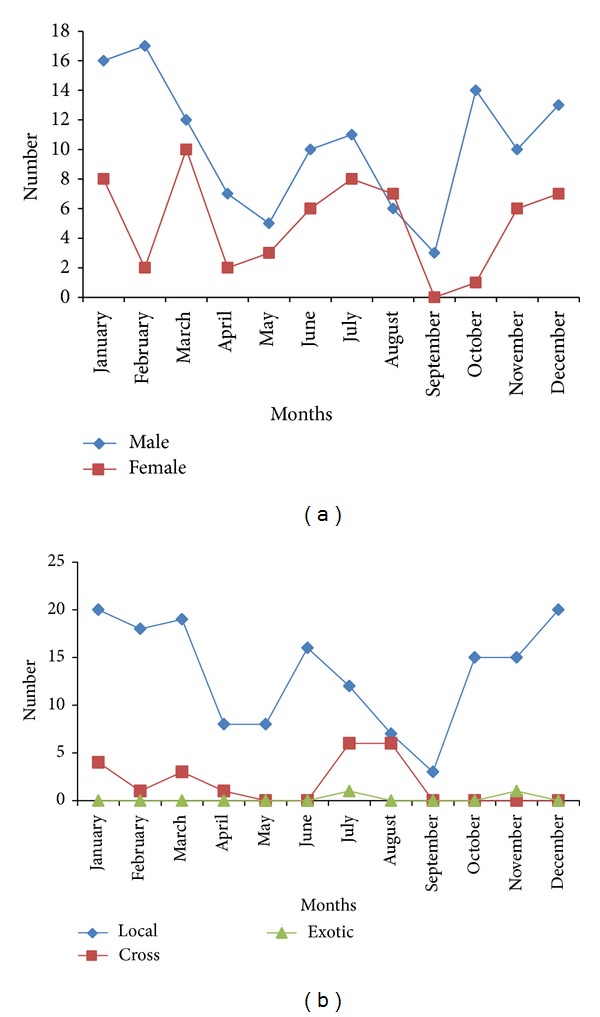
(a) The monthly distribution of Linguatulosis in dogs according to sexes in Jalingo, North Eastern Nigeria, for the year 2010. (b) The monthly distribution of Linguatulosis in dogs according to breeds in Jalingo, North Eastern Nigeria, for the year 2010.

**Figure 4:**
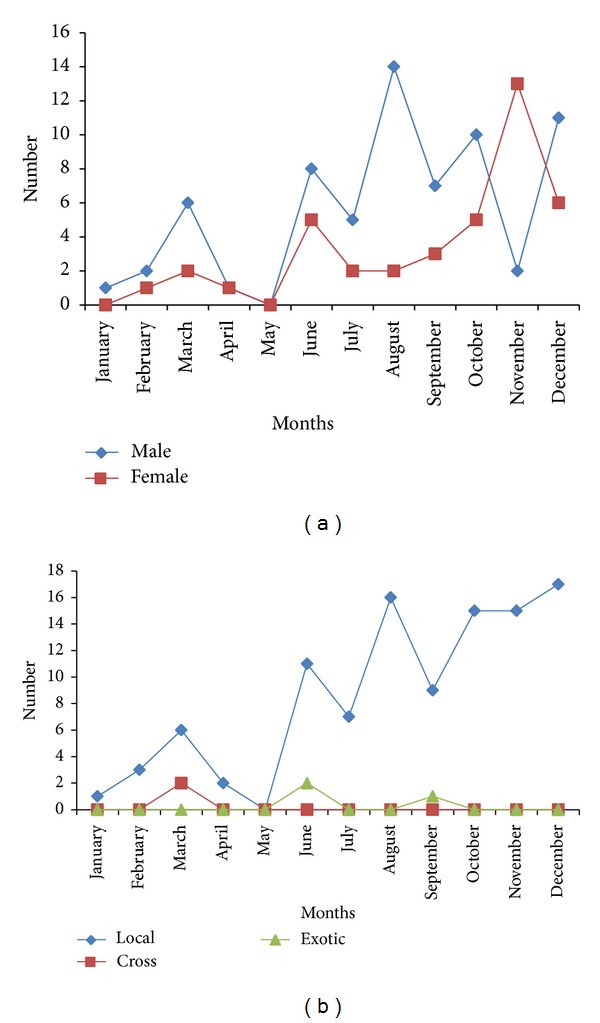
(a) The monthly distribution of Linguatulosis in dogs according to sexes in Jalingo, North Eastern Nigeria, for the year 2011. (b) The monthly distribution of Linguatulosis in dogs according to breeds in Jalingo, North Eastern Nigeria, for the year 2011.

This higher prevalence in the male than in the female was noted throughout the year from January to December in both years (Figures 3(a) and 4(a)). In addition, the prevalence was higher in the local breed of dog than in the cross and exotic throughout the year from January to December (Figures 3(b) and 4(b)).

## 5. Discussion

*Linguatula serrata* is a cosmopolitan species and both larval and nymphal stages have been recorded from humans in Africa, Europe, and the Americas [13]. A prevalence of 38.57% and 35.67% was recorded in 2010 and 2011, respectively, in Jalingo, Taraba State. This is quite higher compared with 25% in Egyptian dogs [14] and 20% in Turkey's dogs [15]. Higher prevalence of 62.2% has been reported by [16] in 2003 in 143 stray dogs in Iran and more recently 76.2% in Shiraz, Iran, by [17]. The dogs were shown to harbor pentastomid parasites in their nasal cavities.

In this study, the parasite was found sublingually. The parasite may have accidentally ensconced itself in the location during migration through the buccal cavity into the nasopharynx. It was also discovered that this aberrant localization is only common in puppies due to the softness of the tissue. In addition, about one-third of dogs were found to be infected with* L. serrata* and the close contact between dogs and livestock may be responsible for the greater rates of infection.

Some locals also believe that eating the raw or undercooked offals, especially liver of farm animals (cattle or sheep), is a useful means to promote the fetus growth during pregnancy because of its high content of iron and vitamins. In the Middle East, Halzoun also occurs after religious feasts in which uncooked sheep or goats may be served [17, 18].

Female dogs showed a lower infection rate in this study than males. This may be linked to the random use of more male animals. The infection was higher in the local breed of dogs than exotic and other crosses. This could be attributed to the fact that the exotic breeds are kept by the rich and affluent people where the animals have better veterinary care and restricted access to outdoor and contact with ruminants or their carcasses. Furthermore, it was discovered that the local (indigenous) breed of dogs were used for security and hunting purposes and, hence, most times are allowed to stray around the streets and farms. Owners also testified to the fact that they often feed their dogs with undercooked offals and bones from the abattoirs and meat markets. Feeding dogs with infected viscera was described to increase the incidence of Linguatulosis in dogs [12]. No significant seasonal variation was noted in the study. However, worthy of note is the slight fall in parasitism observed just before the start of the rainy season (April-May).

With the high prevalence reported in this study the public health implication in a country like Nigeria where the cattle, goats, and sheep meat are quite popular among the local population, the consequences will be far reaching. Taraba State has one of the largest populations of cattle and sheep in Nigeria and the temperate Mambilla plateau where the Canine Linguatulosis was first diagnosed by local vets supplies most of the beef consumed in the southern part of the country.

Byproducts (offal) such as kidney, brain, liver, intestine, heart, and tongue are more commonly consumed by people in impoverished areas of the rural and semiurban regions in the developing countries [19]. Thus, a thorough inspection of visceral organs and particularly lymph nodes should be emphasized in the slaughter house.

In areas, where* L. serrata* is endemic, such as in the Middle East, Visceral Linguatulosis is probably much more common than is generally realized [20]. Eggs, particularly those expelled from infected dogs by sneezing or in nasal secretions, are easily unwittingly ingested as contaminants of food, fingers, water, and formites; hence, veterinarians, dog handlers, and owners could be at risk of the infection and are better advised to wash hands after handling or treating dogs regularly.

The epidemiology of* L. serrata* infections in man is complicated because both eggs and infective larvae can become established. Eggs hatch in the alimentary tract and primary larvae subsequently invade the body cavity to encyst on the viscera, producing Visceral Linguatulosis, whereas ingested infective larvae attempt to migrate to the nasal passages, producing Nasopharyngeal Linguatulosis [21]. Humans could also be infected with the larvae of* L. serrata* by eating raw glands of cattle, sheep, and goats that have the larvae. People may suffer from irritation in their nose and throat. Deaths have been reported due to blocked air passages [22]. Presently, Taraba State seems to be the only state of the federation to have reported the incidence of this disease in all the veterinary hospitals located in the 20 local governments of the states, which defines the potential danger of spread of the disease to all other parts of the country. Most of the clients testified to the fact that they have at one time or the other taken their pets along with them on tourism to the Mambilla Plateau.

In Nigeria, risk factors such as eating undercooked or poorly roasted meat or viscerals, poor hygiene, and vegetative contamination of herbs with dog faeces may also pose danger to humans. Consumption of dog meat and offals as delicacies by some local tribes in Taraba state may also predispose them to this zoonosis. The high prevalence of Linguatulosis in dogs and other animals found in this study highlights the need of improving preventative measures to reduce the rate of infection, which may pose a hazard to human health.

## 6. Conclusion

In conclusion, this study being the first to be done near the Mambilla plateau which is the home for large cattle population due to its favourable weather and good vegetation all year round has established the presence of this parasite in dogs. The high prevalence of Linguatulosis in dogs found in this study highlights the need of improving preventative measures to reduce the rate of infection, which may pose a hazard to human health. We suggest that further investigations about the epidemiology of Linguatulosis in herbivores be conducted in this area.

## Figures and Tables

**Table 1:** The prevalence of *Linguatula serrata* in dogs in Jalingo, North Eastern Nigeria.

Parameters	Examined number	Positive number (%)	Examined number	Positive number (%)
Year	2010		2011	
**Sex**				
Male	301	124 (41.19)	170	67 (39.41)
Female	176	60 (34.09)	130	40 (30.77)
Total	**477**	**184 (38.57)**	**300**	**107 (35.67)**
**Breed**				
Local	350	161 (46.0)	201	102 (50.75)
Cross	100	21 (21.0)	59	2 (3.39)
Exotic	27	2 (7.41)	40	3 (7.5)
Total	**477**	**184 (38.57)**	**300**	**107 (35.67)**
**Age (weeks)**				
0–10	259	130 (50.19)	161	80 (49.69)
11–20	218	54 (24.77)	139	27 (19.42)
Total	**477**	**184 (38.57)**	**300**	**107 (35.67)**
